# Downregulation of macrophage migration inhibitory factor attenuates NLRP3 inflammasome mediated pyroptosis in sepsis-induced AKI

**DOI:** 10.1038/s41420-022-00859-z

**Published:** 2022-02-14

**Authors:** Tianlong Li, Haibin Sun, Yiming Li, Lianjiu Su, Jun Jiang, Ye Liu, Nanhui Jiang, Rong Huang, Jiahao Zhang, Zhiyong Peng

**Affiliations:** 1 Department of Critical Care Medicine, Zhongnan Hospital of Wuhan University, Wuhan, Hubei province 430071 China; 2 Center of Critical Nephrology, Department of Critical Care Medicine, University of Pittsburgh Medical Center, Pittsburgh, PA 15223 USA

**Keywords:** Cell death, Acute kidney injury

## Abstract

Sepsis-induced AKI (acute kidney injury) is considered an inflammation-related disease with high mortality. LPS-induced (Lipopolysaccharide) TLR4-NFκB pathway activation plays an important role in sepsis-induced AKI. Pyroptosis closely associated with inflammation response includes inflammasome formation, caspase1 activation and GSDMD N-terminal fragment cleavage that leads to cell membrane rupture and cell death, which may be related to the pathogenesis of sepsis-induced AKI. MIF (Macrophage migration inhibitory factor), associated with inflammation response, has been proved as a biomarker of sepsis, and perhaps regulate pyroptosis in sepsis-induced AKI. In this study, we focus on investigating the mechanism of MIF promoting pyroptosis in sepsis-induced AKI. MIF and pyroptosis-related proteins were up-regulated in kidney tissue of mice with CLP (cecum ligation puncture) surgery and in LPS-injured human kidney-2 (HK-2) cells. NLRP3 was down-regulated following the suppression of MIF topoisomerase activity by ISO-1 in kidney tissue of CLP mice. Knockdown of MIF alleviated NLRP3 inflammasome mediated pyroptosis in LPS-injured HK-2 cells. Meanwhile, we noted that phosphorylation of p65 was down-regulated by knockdown of MIF. Up-regulation of NLRP3 in response to LPS stimulation could be reversed by JSH-23, an inhibitor of NFκB pathway, but MIF was not affected. In conclusion, up-regulation of MIF in sepsis-induced AKI shows a renal damaged effect that aggravates NLRP3 inflammasome mediated cell pyroptosis through promoting phosphorylation of p65. This study demonstrated a novel mechanism of MIF regulating NLRP3 inflammasome mediated pyroptosis in sepsis-induced AKI.

## Introduction

Sepsis-induced AKI (acute kidney injury) is a high mortality disease related to systemic inflammation reaction [1]. PAMPs (Pathogen associated molecular patterns) and DAMPs (damage associated molecular patterns) are recognized as the dominant causes of sepsis-induced AKI. In response, PRRs (pattern recognition receptors) interact with antigens from PAMPs or DAMPs, recognized rapidly these factors [2]. TLRs (Toll-like receptors) family, especially TLR4, is a most important receptor in sepsis-induced AKI that exists extensively on the cell membrane of renal tubular epithelial cells [3]. Exposed to ligands like LPS (lipopolysaccharide), TLR4-Myd88 mediated phosphorylation of IRAK4 (interleukin-1-receptor-associated kinase) recruits TRAF6 (TNF-receptor-associated factor 6) then interacting with TAK1 complex leading to MAPK (mitogen-activated protein kinase) and NF-κB pathway activation. RelA (p65) was freed from IκB that was then phosphorylated and translocated to the nucleus. NF-κB activation enhances transcription and release of cytokines like TNF-α and IL-1β causing severe inflammation [4]. Continuous stimulation leads to intracellular and extracellular environmental imbalance, followed by infiltration of immune cells, enhance of oxidative stress, and cell death. Kidney injury finally happens involving renal function and morphological change.

Pyroptosis is a cell death related to inflammation. Varieties of inflammasome formation like NLRP1 (nod-like receptor protein 1), NLRP3 (nod-like receptor protein 3), AIM-2 (absent in melanoma 2), NLRC4 (nod-like receptor family CARD domain containing 4) cause cell pyroptosis in inflammatory diseases and cancers. Pyroptosis activators like LPS, ATP (Adenosine Triphosphate) oxidative stress, and potassium outflow lead to disassembly of TGN (trans-Golgi network), followed by PtdIns4P with negative charge recruiting NLRP3, ASC (apoptosis-associated speck like protein containing a CARD) and caspase1 (cysteine-aspartic acid protease 1) to dTGN (dispersed TGN) and finishing the assembly of inflammasome [5]. Cells exposed to LPS, ATP or nigericin appear partial swelling and integrality of the membrane is broken up. In this process, GSDMD (gasdermin D) is cleaved by caspase1 into N- and C- terminal fragments, the former of which punches holes on the membrane. This process matures IL-1β (interleukin-1β) and IL-18 (interleukin-18) and release them extracellular [6]. TLR4-NFκB-NLRP3 pathway has been studied widely in cardiovascular disease and kidney disease, which provides a basis for our study [7, 8].

MIF (Macrophage migration inhibitory factor) is a multi-functional protein with topoisomerase activity, participating in varieties of cell biological activities involving proliferation, metabolism, migration, differentiation and death, extensively existing in marrow-derived macrophages, vascular endothelial cells and tissue epithelial cells [9]. MIF reacts with receptors such as CD44/CD74 and CXCR (chemokine receptors) family and promotes the expression of TLR4 to regulate innate immune [10]. MIF has been considered a necessary factor for NLRP3 inflammasome formation in sepsis and in systemic lupus erythematosus [11, 12]. These studies suggested that MIF might be closely associated with the activation of NLRP3 inflammasome in pyroptosis.

However, there is no evidence about the role of MIF regulating pyroptosis in sepsis-induced AKI. According to previous studies, we hypothesized that up-regulation of MIF in sepsis-induced AKI makes a renal damage effect and promotes NLRP3 inflammasome activation to aggravate cell pyroptosis. In our study, sepsis-induced AKI model was established via mice accepted cecum ligation puncture (CLP) surgery in vivo and HK-2 cells treated with LPS in vitro. We identified that a decline of renal function was related to an up-regulation of MIF and pyroptosis-related proteins in CLP mice. Through a suppression of MIF topoisomerase activity, ISO-1 alleviated renal injury and made NLRP3 expression decrease. To further explore, HK-2 cells were transfected to knockdown MIF and were treated JSH-23 to inhibit p65. Cell morphology was observed under TEM (transmission electron microscope) and cell death was counted via flow cytometry. Finally, LPS-induced pyroptosis modulated by MIF and NF-κB pathway was been proved via immunoblotting. In summary, a solid experimental basis was provided in our study to explore the mechanism of MIF regulating NF-κB-NLRP3 pathway in sepsis-induced AKI.

## Results

### MIF up-regulation, pyroptosis and renal dysfunction were found in CLP mice model

**Fig. 1:**
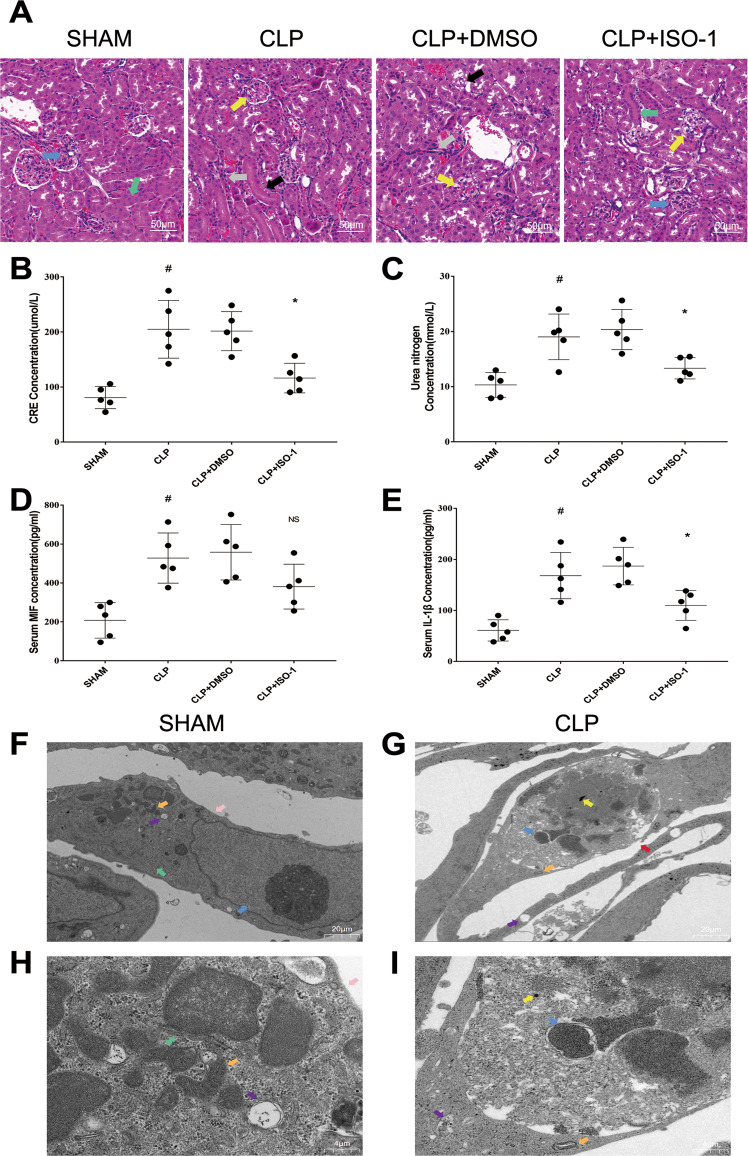
Kidney functions and inflammation of mice in Sham, CLP, CLP + DMSO and CLP + ISO-1 group were assessed through observation of pathological sections, serum creatinine, urea nitrogen, MIF and IL-1β levels. **A** H&E stained pathological sections. Normal glomerulus (blue arrow), normal kidney tubules (green arrow), injured glomerulus (yellow arrow), aggregation of Inflammatory cell (gray arrow), disappear of brushlike margin and vacuolation of renal tubular cells (black arrow). **B**–**E** Creatinine, urea nitrogen, MIF and IL-1β levels in serum. Scale bar = 50 μm. **F**, **G** Morphology of renal tubular epithelial cell in Sham group and in CLP group was observed under TEM (magnification, 2000x). **H**, **I** Orangelles and nucleus of healthy cells in Sham group and in CLP group (magnification, 10,000x). Nucleus (blue arrow), mitochondria (orange arrow), glycogen granules (yellow arrow), endoplasmic reticulum (green arrow), intact cell membrane (pink arrow), broken cell membrane (red arrow), vesicles (purple arrow). Data was mean ± SD from five mice. Each point represented an independent data. Group comparisons were performed by one-way ANOVA followed by Tukey’s post hoc test. (*N* = 5/group, ^#^*P* < 0.05 vs Sham group, *P < 0.05 and **P < 0.01 vs CLP + DMSO group).

**Fig. 2:**
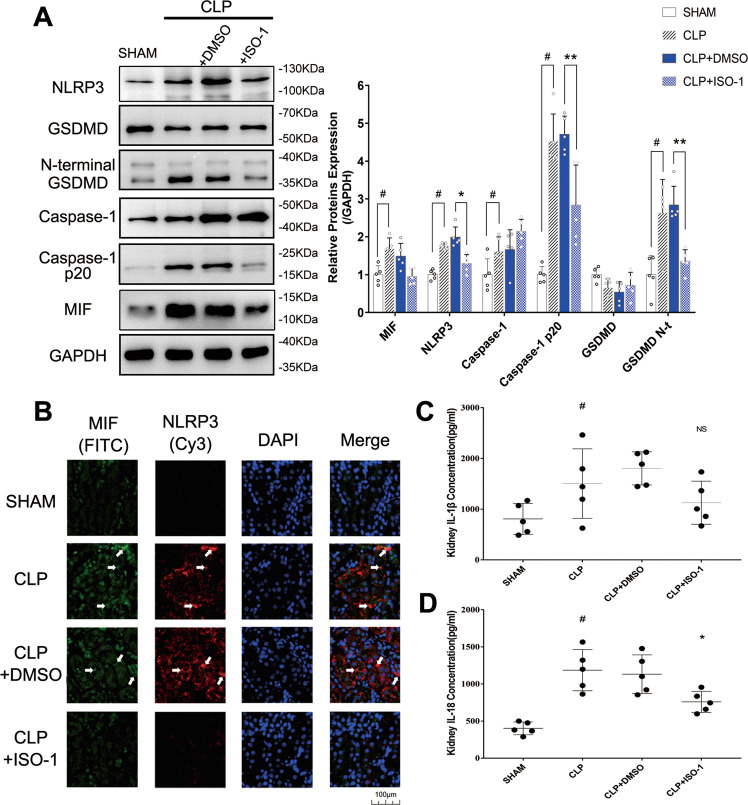
The expression of pyroptosis-related proteins was measured in kidney tissue of mice of Sham, CLP, CLP + DMSO, and CLP + ISO-1 group. **A** MIF, NLRP3, Caspase-1, Caspase-1 p20, Gasdermin D and its N-terminal fragments were measured by western blot. **B** Kidney tissues were stained via immunofluorescence. MIF was tagged by FITC (green) and NLRP3 was tagged by Cy3 (red). The white arrow pointed to the overlapped area of high fluorescence. Scale bar = 100 μm. **C**, **D** IL-1β level and IL-18 level in kidney tissue. Data were mean ± SD from five independent experiments. Each point represented an independent data. Group comparisons were performed by (**A**) two-way ANOVA followed by Tukey’s post hoc test and (**C**) (**D**) one-way ANOVA followed by Tukey’s post hoc test. (*N* = 5/group, ^*#*^*P* < 0.05 vs Sham group, *P < 0.05 and **P < 0.01 vs CLP + DMSO group).

Firstly, worse renal function in CLP mice was confirmed in our study. In contrast with Sham group, the mice in CLP group had worse glomerular and tubular structure via H&E (hematoxylin&eosin) stained kidney tissue sections, involving renal tubular epithelial cell loss, brush edges disappearing and cytoplasmic vacuolation (Fig. 1A). As well as obvious higher levels of creatinine (80.8 μmol/L in sham group vs 204.9 μmol/L in CLP group) and urea nitrogen (10.3 mmol/L in Sham group vs 19.0 mmol/L in CLP group) were detected in serum (Fig. 1B, C). MIF (207.7 pg/ml in sham group vs 528.0 pg/ml in CLP group) and IL-1β (60.9 pg/ml in sham group vs 168.3 pg/ml in CLP group) levels both increased in serum, indicating that severe inflammatory reaction happened in CLP mice (Fig. 1D, E). Renal tubular epithelial cell morphology was observed under TEM, in which cell pyroptosis was found. Compared with Sham group, there existed cell swelling, nucleus pyknosis, glycogen granules increase, endoplasmic reticulum decompose, cell membrane rupture, and indistinct mitochondrial structure in renal tubular epithelial cells of CLP group (Fig. 1F–I). These morphological features are consistent with cell pyroptosis. To further explore the regulation of MIF expression in CLP model, intracellular MIF level was measured through western blot in CLP group, which is 1.68-fold higher than that in Sham group (Fig. 2A). Pyroptosis related proteins in kidney tissue were also measured via ELISA (enzyme-linked immunosorbent assay) or western blot, including NLRP3, GSDMD and its N-terminal fragments, caspase1 (p45 p20), IL-1β (907.4 pg/ml in Sham group vs 1 505 pg/ml in CLP group) and IL-18 (402.2 pg/ml in Sham group vs 1 186 pg/ml in CLP group) in kidney tissue (Fig. 2A–D). NLRP3, caspase1 p20, GSDMD N-terminal fragment and IL-18 were both up-regulated in response to CLP surgery that indicated that cell pyroptosis happened in CLP model. Meanwhile, the result of immunofluorescence demonstrated visually the expression and localization of MIF and NLRP3 in kidney tissue, indicating that MIF might play a role in cell pyroptosis (Fig. 2B).

### ISO-1 alleviated cell pyroptosis in CLP mice model

For exploring the relationship of MIF and pyroptosis in CLP mice model, ISO-1 was used to inhibit MIF topoisomerase activity and reduce inflammatory reaction. CLP + DMSO (dimethylsulfoxide) group as a solvent control group was set up to reduce the experimental error. Renal function was improved in CLP + ISO-1 group contrast with that in CLP + DMSO group, including a reduction of creatinine (201.6 μmol/L in CLP + DMSO group vs 116.3 μmol/L in CLP + ISO-1 group) as well as urea nitrogen (20.4 mmol/L in CLP + DMSO group vs 13.3 mmol/L in CLP + ISO-1 group) in serum, and improved renal tissue morphology visualized in H&E stained sections (Fig. 1A–C). IL-1β (186.9 pg/ml in CLP + DMSO group vs 109.9 pg/ml in CLP + ISO-1 group) in serum were greatly decreased, indicating that ISO-1 attenuated CLP-induced inflammation (Fig. 1D, E). Cell pyroptosis was attenuated that was related to the inhibition of MIF topoisomerase activity, manifested in the decreased expressions of NLRP3, caspase1 p20, GSDMD N-terminal fragment and IL-18 (759 pg/ml in CLP + ISO-1 group vs 1 132 pg/ml in CLP + DMSO group) in kidney tissue (Fig. 2A–C). IL-1β was not down-regulated by ISO-1 in kidney, which meant that IL-1β might lack specificity for pyroptosis in sepsis-induced AKI. We validated the same result via immunofluorescence (Fig. 2B). These results demonstrated that ISO-1 alleviated cell pyroptosis in CLP mice model so that inhibition of MIF topoisomerase activity presented as a renoprotective effect in sepsis-induced AKI.

### LPS mediated up-regulation of MIF and pyroptosis in HK-2 cells

**Fig. 3:**
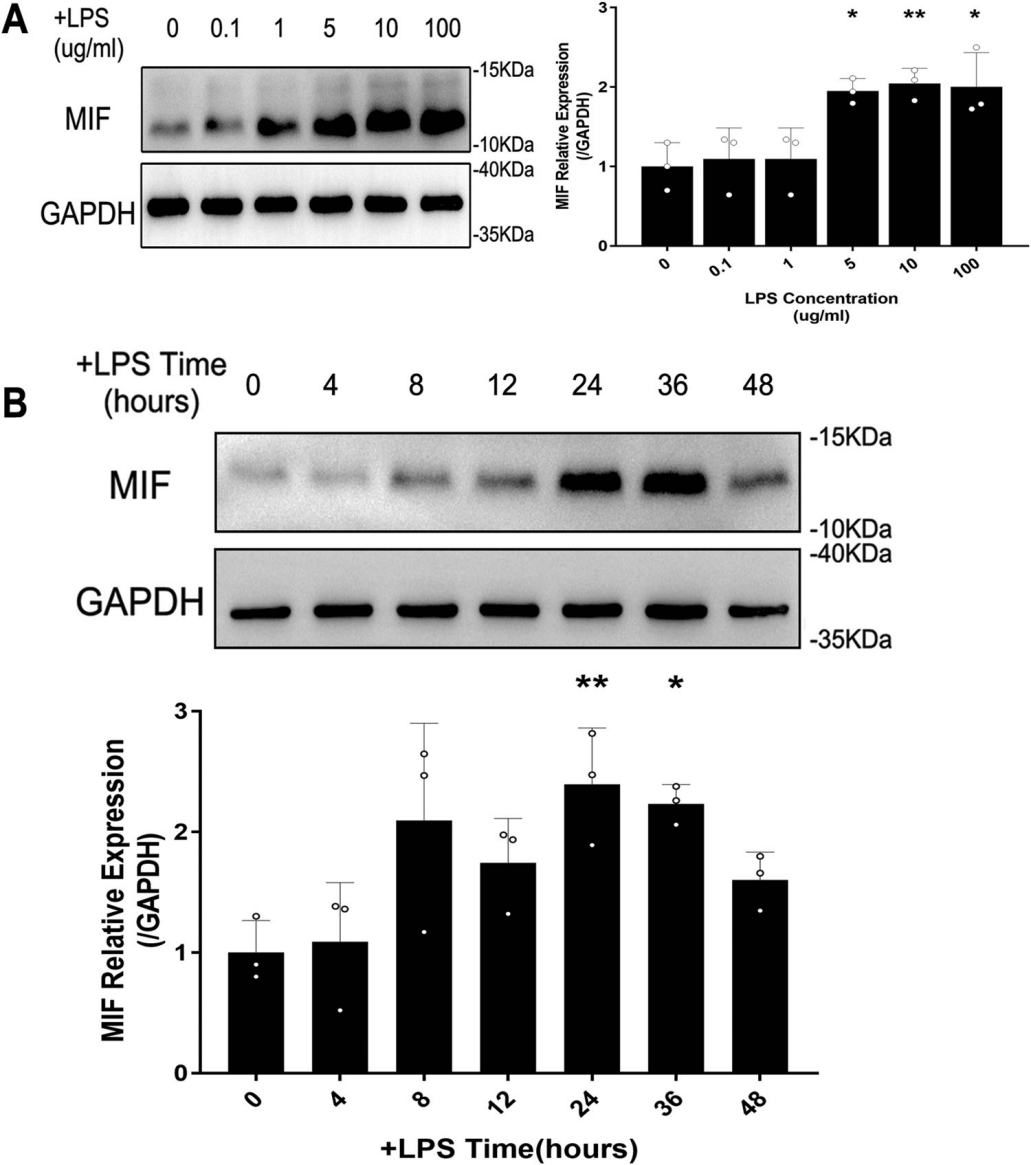
The expression of MIF was measured in HK-2 cells at different LPS concentrations and time points. **A** MIF expression in HK-2 cells treated by 0, 0.1, 1, 5, 10, 100 μg/ml LPS for 12 h. **B** MIF expression at 10 ug/ml LPS concentration for 0, 4, 8, 12, 24, 36, 48 h. Data were mean ± SD from three independent experiments. Each point represented an independent data. Group comparisons were performed by one-way ANOVA followed by Tukey’s post hoc test. (*N* = 3/group, *P <0.05 and **P <0.01 vs 0 μg/ml LPS dose group or 0 h +LPS group).

**Fig. 4:**
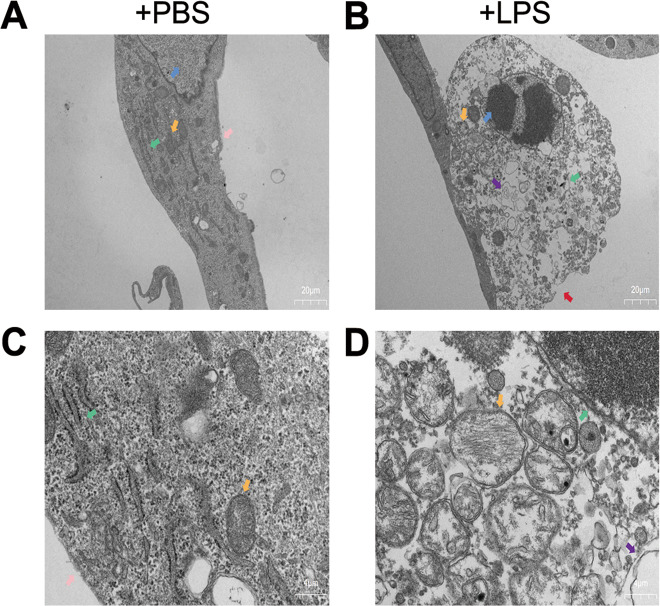
Morphology of HK-2 cells was observed under the transmission electron microscope. **A**, **B** HK-2 cells were treated with PBS or 10 μg/ml LPS for 24 h (magnification, 2000x). **C**, **D** Organelles of normal HK-2 cells or LPS-injured HK-2 cells (magnification, 10,000x). Nucleus (blue arrow), mitochondria (orange arrow), endoplasmic reticulum (green arrow), intact cell membrane (pink arrow), broken cell membrane (red arrow), vesicles (purple arrow).

In vitro, intracellular MIF level was measured to select the appropriate LPS dose and treatment time. Based on preliminary experiment, HK-2 cells were treated with different LPS concentration (0, 0.1, 1, 5, 10, 100 μg/ml) for 12 h. The peak level of MIF appeared at 10 μg/ml LPS stimulation (Fig. 3A). On this LPS concentration, we detected MIF level via western blot in different time points (0, 4, 8, 12, 24, 36, 48 h, Fig. 3B). Thus, the condition of 10 μg/ml LPS stimulation for 24 h was suitable in vitro experiment. An obvious change of morphology in LPS-injured HK-2 cells contrast with negative control cells were observed directly under TEM, including nucleus pyknosis, intracellular vesicle formation, cell membrane rupture and mitochondrial crest fracture (Fig. 4A–D). The results suggested that up-regulation of MIF in HK-2 cells and their pyroptosis were induced by LPS.

### MIF knockdown reduced pyroptosis and suppressed NF-κB pathway in LPS-injured HK-2 cells

**Fig. 5:**
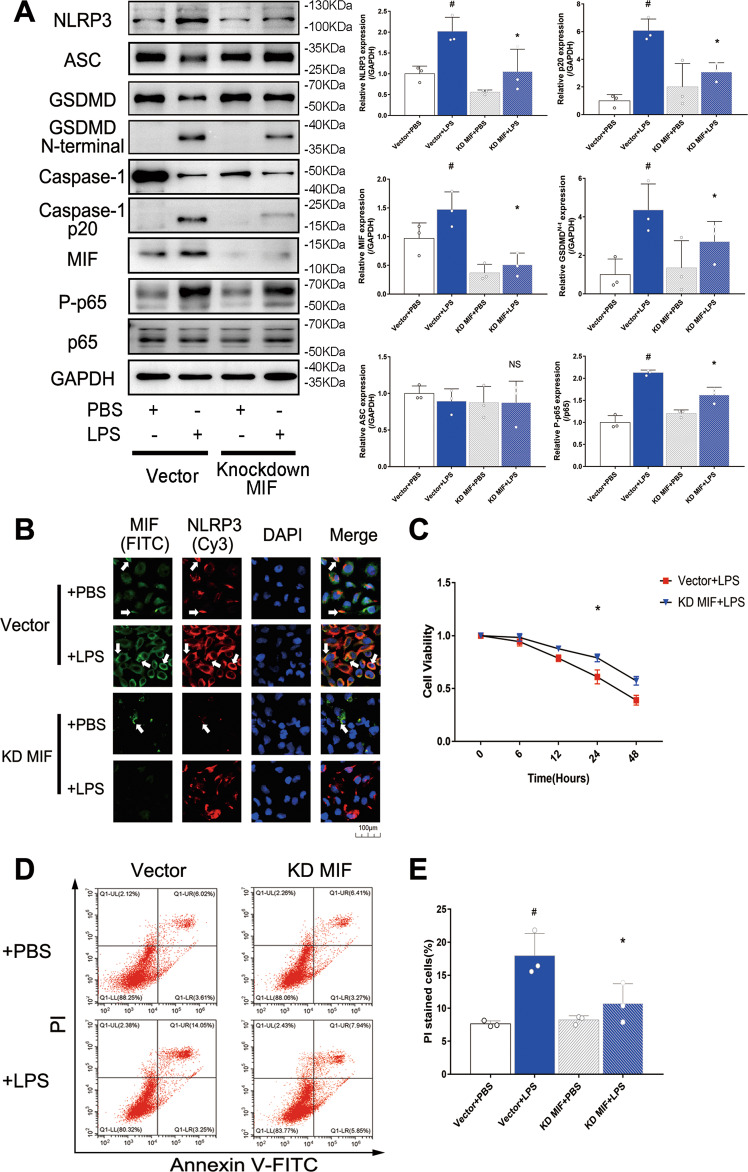
The relationship of MIF, NF-κB and pyroptosis in HK-2 cells of vector+PBS, vector+LPS, knockdown MIF + PBS and knockdown MIF + LPS group. **A** MIF, pyroptosis related proteins (NLRP3, ASC, Caspase-1, Caspase-1 p20, Gasdermin D and its N-terminal fragment), p65 and phosphorylated p65 were detected, and their expressions relative to GAPDH were shown. **B** HK-2 cells were stained via immunofluorescence. MIF was tagged by FITC (green) and NLRP3 was tagged by Cy3 (red). The white arrow pointed to the overlapped area of high fluorescence. Scale bar = 100 μm. **C** Cell viability of HK-2 cells was evaluated via CCK-8 method. **D** HK-2 cells were stained by annexin-V-FITC and propidium iodide(PI). **E** PI stained HK-2 cells were counted by flow cytometry. Data were mean ± SD from three independent experiments. Each point represented an independent data. Group comparisons were performed by (**A**) & (**E**) one-way ANOVA followed by Tukey’s post hoc test, and (**C**) two-way ANOVA followed by Tukey’s post hoc test. (*N* = 3/group, ^#^*P* < 0.05 vs vector + PBS group, *P < 0.05 vs vector + LPS group, NS means no significant difference).

To further explore the mechanism of MIF promoting pyroptosis in HK-2 cells, MIF gene was inhibited by lentivirus to suppress MIF protein expression, which was verified via western blot (Fig. 5A). NLRP3 expression was down-regulated in knockdown MIF group that was measured via immunoblotting and immunofluorescence (Fig. 5A, B). Consistently, caspase1 p20 and GSDMD N-terminal fragment were both down-regulated that were measured via immunoblotting (Fig. 5A). However, ASC protein, as a composition of NLRP3 inflammasome, was not significantly changed, which demonstrated that MIF could not cause ASC-mediated cell death (Fig. 5A). Next, proliferation and death of HK-2 cells were evaluated respectively via CCK-8 method and flow cytometry. HK-2 cells in LPS group presented a slow proliferation rate over time, but this effect was obviously alleviated by knockdown of MIF (Fig. 5C). PI stained HK-2 cells counting was also reduced. (17.9% in vector +LPS group vs 10.7% in Knockdown MIF + LPS group, Fig. 5D, E) The results suggested that MIF knockdown reduced cell pyroptosis via down-regulated NLRP3 in LPS-injured HK-2 cells.

### MIF promoted phosphorylation of p65 to aggravate NLRP3 mediated pyroptosis

**Fig. 6:**
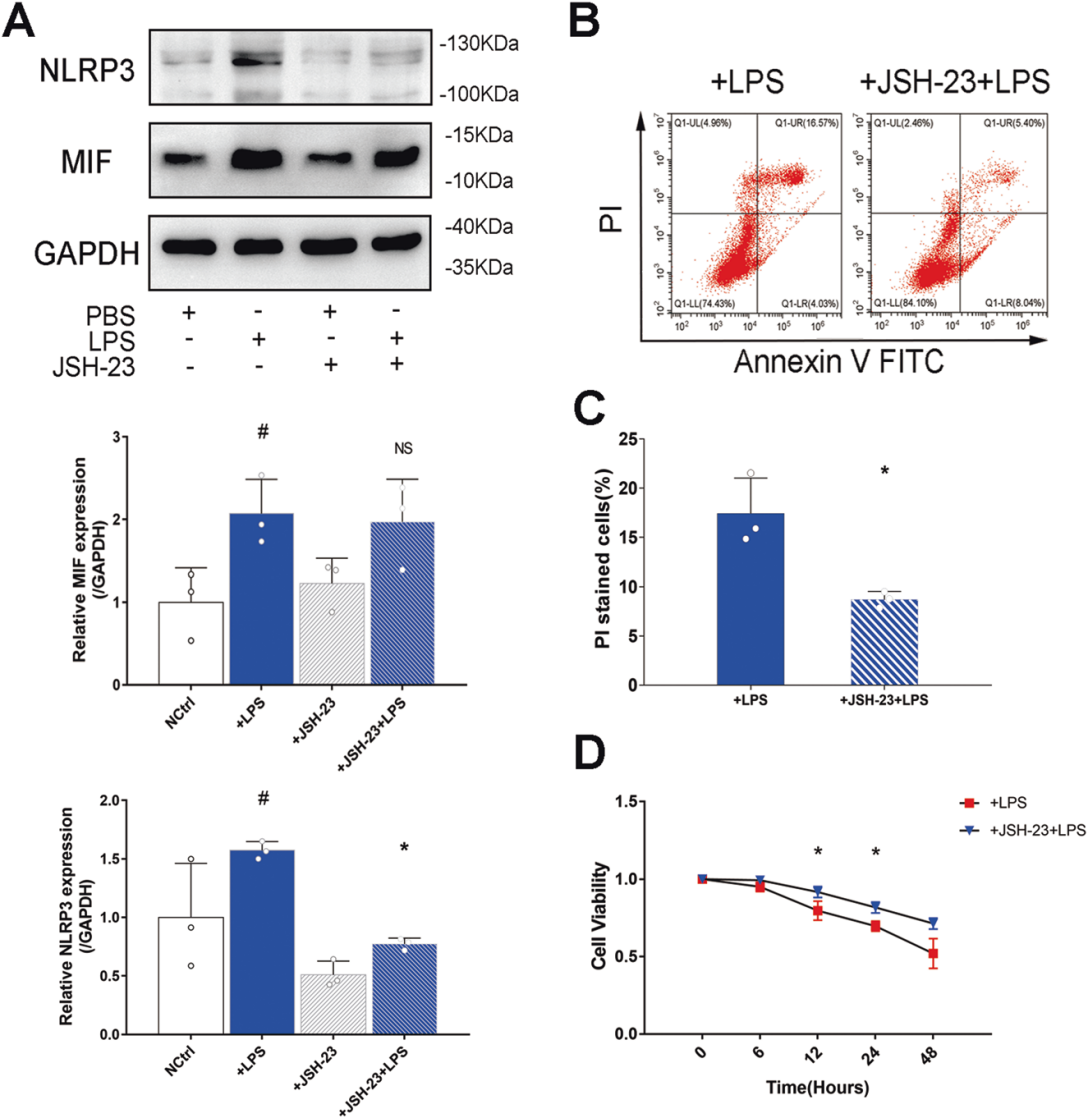
the mechanism of MIF promoting pyroptosis was verified via JSH-23 inhibiting p65 transfer to the nucleus. **A** MIF and NLRP3 expressions relative to GAPDH were measured by western blot. **B** HK-2 cells of +LPS and +JSH-23+LPS group were stained by annexin-V-FITC and PI. **C** PI stained cells were counted by flow cytometry. **D** Cell viability of HK-2 cells was evaluated via CCK-8 method. Data were mean ± SD from three independent experiments. Each point represented an independent data. Group comparisons were performed by (**A**) one-way ANOVA followed by Tukey’s post hoc test, (**C**) unpaired *T*-test and (**D**) two-way ANOVA followed by Tukey’s post hoc test. (*N* = 3/group, ^#^*P* < 0.05 vs +PBS group, *P < 0.05 vs +LPS group, NS means no significant difference).

Existing research about NF-κB-NLRP3 pathway inspired our study. To investigate the mechanism of MIF regulating NLRP3 in LPS-injured HK-2 cells, p65 and P-p65 (phosphorylated-p65) expressions were measured based on knockdown MIF. P-p65 level was increased in HK-2 cells treated with LPS, but this situation was reversed by knockdown MIF (Fig. 5A). To further confirm this result, JSH-23, an inhibitor of p65, was used to reduce p65 concentration in the nucleus. NLRP3 was obviously down-regulated in LPS-injured HK-2 cells treated with JSH-23, but MIF expression was not affected (Fig. 6A). Through inhibition of NF-κB pathway, PI stained HK-2 cells counting was reduced.(17.4% in +LPS group vs 8.7% in +JSH-23+LPS group, Fig. 6B, C).These results meant that MIF promoted phosphorylation of p65 to enhance downstream transcription of NF-κB pathway and finally activated NLRP3 inflammasome.

## Discussion

AKI is a common and complicated disease in clinical practice. Approximately 50% of AKI is complicated by sepsis, with high mortality [13]. Inflammatory reaction, oxidative stress and cell damage are among involved in the development of sepsis-induced AKI. Inflammatory factors are recognized as an important role in PAMPs&DAMPs of sepsis-induced AKI. Substantial IL-1β, IL-6, TNF-α, and cell fragments cause renal tubular epithelial cell damage in sepsis-induced AKI [3]. Therefore, cell death and its mechanism become the focus of DAMPs in AKI. Based on recent studies, pyroptosis and apoptosis may be the vital cell death way in sepsis-induced AKI. Pyroptosis is a cell death way associated with inflammation that has been extensively studied. NLRP3 inflammasome mediated pyroptosis was confirmed as an important mechanism of diabetes [14]. In Parkinson’s disease, TLR4-NFκB pathway activation and potassium outflow both promoted pyroptosis of neuronal cells [15]. In a previous study, we found interestingly that caspase1 mediated cell death and caspase3 mediated cell death both happened in vivo [16]. Caspase1 plays a dual function in two cell death way under certain conditions [17]. These studies suggest that there may be crosstalk of pyroptosis and apoptosis in sepsis-induced AKI. In addition, Caspase4/5/11, as a key factor of the non-classical pathway of pyroptosis [18], is worthy of being studied further in sepsis-induced AKI in the future. In our study, we demonstrated that NLRP3, caspase1 p20 and GSDMD N-terminal were up-regulated in sepsis-induced AKI. Compared with that in normal HK-2 cells, obvious morphological changes in LPS-injured HK-2 cells were observed under TEM, including cell swelling, cell membrane rupture, vesicles formation, and mitochondrial disorder. These results suggested that NLRP3 inflammasome mediated pyroptosis made a renal damage effect in this disease model.

MIF is a pro-inflammatory cytokine widely expressed by bone marrow-derived macrophages, vascular endothelial cells, tissue epithelial cells and some tumor cells [9]. MIF modulates TLR4-mediated innate immunity that recruits immune cells to gather in inflammatory areas, activates inflammatory pathways, and induces immune cell differentiation. The pro-inflammatory ability of MIF is closely associated with receptor CD44/CD74 and CXCR family [19, 20]. Characteristics of MIF make it a potential biomarker of sepsis [21]. Consequently, the mechanism of MIF regulating cell death is gradually revealed. MIF prevents p53 translocation from cytoplasm to nucleus and inhibits p53-induced apoptosis and cell cycle arrest [22]. MIF can also inhibit the release of cytochrome c from mitochondria via suppressing Bim-induced apoptosis [23]. Interestingly, MIF has an opposite function in pyroptosis. Exogenous MIF and endogenous MIF both could promote NLRP3 inflammasome formation [11, 12]. To further explore the mechanism of MIF mediated pyroptosis in sepsis-induced AKI, ISO-1 was used to suppress MIF topoisomerase activity in CLP mice, and knockdown of MIF was achieved by lentivirus in LPS-injured HK-2 cells. NLRP3, caspase-1 p20 and GSDMD N-terminal fragment expressions were reversed by down-regulation of MIF. These suggested that inhibition of MIF alleviated NLRP3 inflammasome mediated pyroptosis in sepsis-induced AKI.

NFκB-NLRP3 pathway is considered an important mechanism in inflammatory diseases. TLR4-NFκB activation amplifies the neuroinflammatory response induced by ethanol through regulating NLRP3 inflammasome formation [24]. The decreased expressions of NF-κB and NLRP3 induced by mitophagy activation alleviate acetaminophen-induced acute liver injury [25]. In addition, MIF can enhance downstream transcription of NF-κB pathway via interacting with TXNIP (thioredoxin-interacting protein) [26]. Based on these studies, we confirmed that MIF promoted phosphorylation of p65, and then tried to inhibit NF-κB pathway by JSH-23. NLRP3 expression was decreased by JSH-23, but MIF was not affected. Taken together, MIF promoted NLRP3 inflammasome mediated cell pyroptosis in sepsis-induced AKI via modulating NF-κB pathway.

Our study has numerous strengths. The chief strength is that the conclusion was confirmed from CLP mice model and HK-2 cells line (Two species). As far as we know, our study is the first one to reveal the mechanism of MIF promoting pyroptosis in sepsis-induced AKI. We used LPS as the only stimulator in vitro to make sure that the disease model was consistent with in vivo. Moreover, knockdown of MIF was used to prove the relationship between MIF and pyroptosis that made the conclusion more credible. However, some limitations are also existing in our study. Firstly, knockout mice were not used in vivo because of time and financial constraints. Secondly, there was no specific method to detect pyroptosis, so that we could only determine the occurrence of pyroptosis in our study through PI stained cells, morphology under TEM and pyroptosis related proteins. Thirdly, as for images of TEM, we could not distinguish morphological changes induced by knockdown of MIF, so that images of knockdown MIF group were not shown. Although the role of MIF regulating NLRP3 inflammasome mediated pyroptosis in sepsis-induced AKI has been confirmed by our study, further studies will be continued in the future.

**Fig. 7:**
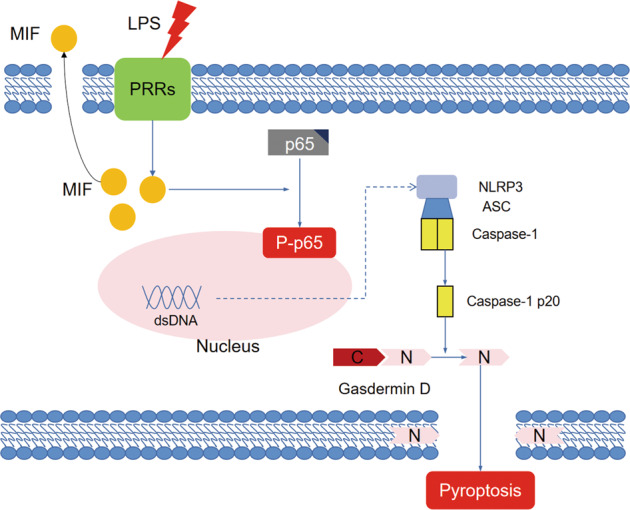
Mechanism diagram of MIF/NF-κB-NLRP3 pathway in sepsis-induced AKI. The mechanism of MIF promoting pyroptosis of kidney tubular cells through regulating NF-κB-NLRP3 pathway in sepsis-induced AKI.

In conclusion, MIF plays an effect of renal damage in sepsis-induced AKI, up-regulation of MIF in response to LPS stimulation promotes phosphorylation of p65 and finally aggravated NLRP3 inflammasome mediated cell pyroptosis (Fig. 7).

## Materials and methods

### Mice model of AKI

Mice feeding and model building were performed in ABSL-3 Lab (animal biosafety level-3 laboratory) of Wuhan University. A total of twenty C57BL/6 male mice were purchased from Vital River Laboratory Animal Technology Co. Ltd (Beijing, China). Based on previous study, the number of mice was appropriate for our study. CLP surgery was performed strictly as described in the literature [27]. The mice (25–30 g weight, 12–16 weeks old) that met the experimental requirements were simply and randomly selected and assigned to Sham group, CLP group, CLP + DMSO group and CLP + ISO-1 group. Each mouse was numbered to achieve single blind. After mice were anesthetized with isoflurane, a median incision (1.5–2.0 cm) was made below the diaphragm to expose the cecum. A ligation was made with a 5–0 silk ligature suture at the juncture of the colon and cecum. The cecum was punched twice with a 22-gauge needle and feces were squeezed out a little. Finally, the peritoneum and skin were sutured respectively with a 5–0 silk ligature suture and each mouse with CLP surgery was fluid-resuscitated with preheated saline (37 °C, 50 μl/g body weight) subcutaneously. Mice in Sham group accepted median incision but not cecum ligation puncture. ISO-1 ((S,R)-3-(4-hydroxyphenyl)-4,5-dihydro-5-isoxazole acetic acid methyl ester) has a significant effect on inhibiting MIF topoisomerase activity, which suppresses inflammatory response in sepsis [28]. ISO-1 (Selleck Chemicals, Houston, Texas, USA) was dissolved into 1% DMSO and each mouse in CLP + DMSO group and CLP + ISO-1 group was injected intraperitoneally according to body weight (ISO-1 20 μg/g body weight and the same amount of 1% DMSO as control group) within 4–6 h before CLP surgery and every 12 h after. As for samples, the numbers were tagged on the tubes to ensure examiners blinded. Heparinized blood was centrifuged in 1500 r/min, 4 °C to separate the serum that was then stored in −80 °C for further measurements. Two kidneys of each mouse were harvested, the left kidney was immersed in 4% paraformaldehyde for H&E staining and the right kidney was frozen by liquid nitrogen and stored in −80 °C for protein detection. In vivo experiments were performed by Chinese legislation on the use and care of laboratory animals and approved by the Animal Care and Use Committee of Wuhan University.

### Evaluation of renal function and histology

Creatinine and urea nitrogen was measured to evaluate renal function via commercialized kit reagents (Jiancheng Bioengineering Institute, Nanjing, China). The absorbance at 546 nm was set to measure the level of creatinine and urea nitrogen via a multimode plate reader (Enspire, PerkinElmer, Massachusetts, USA). Kidney tissue fixed by formalin and embedded by paraffin was cut into 3 μm slices and visualized under an optical microscope.

### Cell culture

HK-2 cells line was purchased from and identified by Cell Bank of Chinese Academy of Sciences (Shanghai, China). The cells were identified by short tandem repeat and tested negative for mycoplasma before being used in vitro experiments. We tested HK-2 cells every month to make sure they were not contaminated with mycoplasma. Cultured in Minimum Essential Medium (MEM, 01-026-1ACS, Biological Industries, Kibbutz Beit Haemek, Israel) with 10% fetal bovine serum (FBS, 04-010-1 A, Biological Industries, Kibbutz Beit Haemek, Israel) and 1% penicillin-streptomycin solution, HK-2 cells in 10 cm culture dish were incubated in 37 °C, 5% CO2. As at a density of 80% or more, HK-2 cells would be subcultured. HK-2 cells were carefully counted and seeded into cell plates before being used for experiments. LPS (Ultrapure LPS, E. coli 0111:B4, InvivoGen, California, USA) was dissolved in PBS solution and then stored at −20 °C for subsequent experiments. Before being treated with LPS or PBS, HK-2 cells were washed with PBS and treated with serum-free MEM medium 6 h in advance. HK-2 cells were then treated with serum-free MEM medium containing 10 μg/ml LPS or same volume PBS solution and incubated in 37 °C, 5% CO2. HK-2 cells were pre-incubated for 3 h with JSH-23 (100 μM/L) to inhibit p65 translocation to the nucleus [29, 30].

### Lentivirus transfection

Custom-made recombinant lentivirus and its vector were designed and purchased from GenePharma Corporation (Shanghai, China). Through inhibiting the transcription of MIF gene by lentivirus, knockdown of MIF protein was achieved. Lentivirus (Sequences from 5′ to 3′ -TGGACAGGGTCTACATCAACTTCAAGAGAGTTGATGTAGACCCTGTCCTTTTTTC-) and its vector (Sequences from 5′ to 3′ -CACCGTTCTCCGAACGTGTCACGTCAAGAGATTACGTGACACGTTCGGAGAATTTTTTG-) carried anti-puromycin resistance gene sequences. HK-2 cells at a density of 20-30% were added with MEM medium with approximately 10^7 ^V.G. /ml lentivirus solution and then incubated at 37 °C for 2–3 days. After the cell density reached 80%, puromycin was used to select HK-2 cells with puromycin-resistant phenotype, and the medium was changed every 24 h. Until cell density reaching 80% again, HK-2 cells with knockdown of MIF protein were gotten. MIF levels were then measured via PCR (polymerase chain reaction) and western blot to ensure that the lentivirus could inhibit MIF expression.

### ELISA

Samples were thawed at room temperature. Based on the preliminary experiment, serum samples did not need to be diluent, and proteins in kidney tissue were extracted via lysis buffer. MIF (KE10027, Protein Tech, Wuhan, China), IL-1β (KE10003, Protein Tech, Wuhan, China) and IL-18 (ab216165, Abcam, Cambridgeshire, UK) levels were measured with a commercialized sandwich enzyme-linked kit following the manufacturer’s instructions. Each sample was measured in triplicate.

### Western blot

The samples added lysis buffer were incubated on ice for 15 min and centrifuged at 12,000 r/min. The supernatant was then gotten. After measuring the protein concentration in the samples via the bicinchoninic acid method, the samples were mixed with sodium dodecyl sulfate loading buffer and boiled for 15 min. Equal amounts of proteins were separated by sodium dodecyl sulfate-polyacrylamide gel electrophoresis and transferred to a polyvinylidene fluoride membrane. Blocking with 5% skimmed milk solution or 5% bovine serum albumin solution, the membrane was cut into suitable strips and incubated with primary antibodies of MIF (88186, Cell Signaling Tech, Boston, Massachusetts, USA), NLRP3 (DF7438, Affinity Biosciences, Jiangsu, China), ASC (DF6304, Affinity Biosciences, Jiangsu, China), P-p65 (AF2006, Affinity Biosciences, Jiangsu, China), p65 (AF5006, Affinity Biosciences, Jiangsu, China), Caspase-1 (p45, p20) (22915-1-AP, Protein Tech, Wuhan, China), GAPDH (60004-1-Ig, Protein Tech, Wuhan, China), GSDMD and its N-terminal fragment (AF4012, Affinity Biosciences, Jiangsu, China) at 4 °C overnight. The strips of membrane were washed by 1× TBST solution and then incubated secondary antibodies. The bands of proteins were developed by an electrochemiluminescence imaging system (Tanon-5200, Shanghai, China).

### Immunofluorescence

2.5 cm sterile coverslips were placed in a six-well plate. At a density of 3 × 10^5^ /well HK-2 cells were seeded on the coverslips to make them grow consistently. After the density of cells reached over 70%, the cells were treated with LPS or equivalent amount of PBS and incubated at 37 °C for 24 h. HK-2 cells were then washed with PBS solution and fixed with 4% paraformaldehyde. The fixed cells were incubated overnight with MIF (20415-1-AP, Protein Tech, Wuhan, China) and NLRP3 (DF7438, Affinity Biosciences, Jiangsu, China) primary antibodies solution at 4 °C. MIF and NLRP3 were labeled with FITC (green, fluorescein isothiocyanate) and Cy3 (red, cyanine3) fluorescent antibodies, respectively, and the nucleus were labeled with DAPI (4′,6-diamidino-2-phenylindole). The images were observed under a fluorescence microscope (IX73, Olympus, Tokyo, Japan). In the same way, the slices of kidney tissue fixed by formalin and embedded by paraffin were incubated with MIF and NLRP3 antibodies.

### Transmission electron microscope (TEM)

Kidney tissues or HK-2 cells fixed by 2.5% glutaraldehyde were scraped into tubes and stored at 4 °C. The samples were washed and refixed with osmium tetroxide solution, made as nanoscale slices, stained with uranium acetate and lead citrate, respectively, washed with distilled water and then observed under the electron microscope (Tecnai, FEI, Oregon, USA). Single-cell morphology was observed under 1000–2000× magnification, as for Organelles and nuclei under 5000–10,000× magnification. The characteristics of pyroptosis we found in our study were based on descriptions in the literature [31].

### Cell viability

HK-2 cells of lentivirus transfected groups and their negative controls at a density of 5 × 10^3^ cells/well with PBS or LPS were seeded into 96-well plate for 0–48 h. Cell counting kit-8 (CCK-8, HY-K0301, MedChemExpress, New Jersey, USA) was used to evaluate cell viability. The absorbance was detected under sterile conditions at 450 nm via a multimode plate reader at each point time. The absorbance at 0 h had been normalized and considered as a control group, which was compared with the absorbance of other groups. Cell viability was the ratio of the two groups absorbance.

### Flow cytometry

HK-2 cells of various groups were seeded into six-well plates, and PBS or LPS was added into cell culture after the density reached over 70%. The cells were incubated at 37 °C for 24 h. Cell death was detected by a commercialized apoptosis detection kit (C1062M, Beyotime, Shanghai, China). Cells were suspended by ethylenediamine tetraacetic acid-free trypsin, and incubated with annexin-V-FITC (fluorescein isothiocyanate) and PI (propidium Iodide) for flow cytometry. The counting of PI stained cells reflected cell death due to cell membrane rupture, but could not specifically represent cell pyroptosis.

### Statistical analysis

Data including at least three times independent experiments (biological replicates) were presented as mean ± SD (standard deviation) and were analyzed by Graphpad Prism 7.0 (San Diego, California, USA). Each independent data was showed as a point in graphs. Unpaired *T*-test was used for two-group comparisons in counting PI stained cells (Fig. 6C). One-way analysis of variance (ANOVA) followed by the Tukey’s post hoc tests was used for multiple-group comparisons of one independent factor. Two-way ANOVA followed by the Tukey’s post hoc tests was used for multiple-group comparisons of multiple independent factors. *P*-value less than 0.05 was considered a statistically significant difference.

## Supplementary information

Uncropped Western blots

Reproducibility checklist

## Data Availability

The datasets used and analyzed during the current study are available from the corresponding author on reasonable request.

## References

[1] Hoste EA, Bagshaw SM, Bellomo R, Cely CM, Colman R, Cruz DN (2015). Epidemiology of acute kidney injury in critically ill patients: the multinational AKI-EPI study. Intensive Care Med.

[2] Peerapornratana S, Manrique-Caballero CL, Gómez H, Kellum JA (2019). Acute kidney injury from sepsis: current concepts, epidemiology, pathophysiology, prevention and treatment. Kidney Int.

[3] Gomez H, Ince C, De Backer D, Pickkers P, Payen D, Hotchkiss J (2014). A unified theory of sepsis-induced acute kidney injury: inflammation, microcirculatory dysfunction, bioenergetics, and the tubular cell adaptation to injury. Shock.

[4] Vázquez-Carballo C, Guerrero-Hue M, García-Caballero C, Rayego-Mateos S, Opazo-Ríos L, Morgado-Pascual JL (2021). Toll-like receptors in acute kidney injury. Int J Mol Sci.

[5] Chen J, Chen ZJ (2018). PtdIns4P on dispersed trans-Golgi network mediates NLRP3 inflammasome activation. Nature.

[6] Shi J, Zhao Y, Wang K, Shi X, Wang Y, Huang H (2015). Cleavage of GSDMD by inflammatory caspases determines pyroptotic cell death. Nature.

[7] Huang Z, Zhuang X, Xie C, Hu X, Dong X, Guo Y (2016). Exogenous hydrogen sulfide attenuates high glucoseinduced cardiotoxicity by inhibiting NLRP3 inflammasome activation by suppressing TLR4/NF-κB pathway in H9c2 cells. Cell Physiol Biochem.

[8] Sharawy M, Serrya M (2020). Pirfenidone attenuates gentamicin-induced acute kidney injury by inhibiting inflammasomedependent NLRP3 pathway in rats. Life Sci.

[9] Kang I, Bucala R (2019). The immunobiology of MIF: function, genetics and prospects for precision medicine. Nat Rev Rheumatol.

[10] Jankauskas SS, Wong DWL, Bucala R, Djudjaj S, Boor P (2019). Evolving complexity of MIF signaling. Cell Signal.

[11] Lang T, Lee JPW, Elgass K, Pinar AA, Tate MD, Aitken EH (2018). Macrophage migration inhibitory factor is required for NLRP3 inflammasome activation. Nat Commun.

[12] Shin MS, Kang Y, Wahl ER, Park HJ, Lazova R, Leng L (2019). Macrophage migration inhibitory factor regulates U1 small nuclear RNP immune complex-mediated activation of the NLRP3 inflammasome. Arthritis Rheumatol.

[13] Zarjou A, Agarwal A (2011). Sepsis and acute kidney injury. J Am Soc Nephrol.

[14] Qiu Z, Lei S, Zhao B, Wu Y, Su W, Liu M et al. NLRP3 inflammasome activation-mediated pyroptosis aggravates myocardial ischemia/reperfusion injury in diabetic rats. Oxid Med Cell Longev. 2017. 10.1155/2017/9743280. 10.1155/2017/9743280 PMC5618779 29062465

[15] Wang S, Yuan YH, Chen NH, Wang HB (2019). The mechanisms of NLRP3 inflammasome/pyroptosis activation and their role in Parkinson’s disease. Int Immunopharmacol.

[16] Li YM, Zhang J, Su LJ, Kellum JA, Peng ZY (2019). Downregulation of TIMP2 attenuates sepsis-induced AKI through the NF-κb pathway. Biochim Biophys Acta Mol Basis Dis.

[17] Tsuchiya K, Nakajima S, Hosojima S, Thi Nguyen D, Hattori T, Manh Le T (2019). Caspase-1 initiates apoptosis in the absence of gasdermin D. Nat Commun.

[18] Kayagaki N, Stowe IB, Lee BL, O’Rourke K, Anderson K, Warming S (2015). Caspase-11 cleaves gasdermin D for non-canonical inflammasome signalling. Nature.

[19] Roger T, David J, Glauser MP, Calandra T (2001). MIF regulates innate immune responses through modulation of Toll-like receptor 4. Nature.

[20] Calandra T, Roger T (2003). Macrophage migration inhibitory factor: a regulator of innate immunity. Nat Rev Immunol.

[21] Pohl J, Papathanasiou M, Heisler M, Stock P, Kelm M, Hendgen-Cotta UB (2016). Renal replacement therapy neutralizes elevated MIF levels in septic shock. J Intensive Care.

[22] Jung H, Seong HA, Ha H (2008). Critical role of cysteine residue 81 of macrophage migration inhibitory factor (MIF) in MIF-induced inhibition of p53 activity. J Biol Chem.

[23] Liu L, Chen J, Ji C, Zhang J, Sun J, Li Y (2008). Macrophage migration inhibitory factor (MIF) interacts with Bim and inhibits Bim-mediated apoptosis. Mol Cells.

[24] Ibáñez F, Montesinos J, Ureña-Peralta JR, Guerri C, Pascual M (2019). TLR4 participates in the transmission of ethanol-induced neuroinflammation via astrocyte-derived extracellular vesicles. J Neuroinflammation.

[25] Shan S, Shen Z, Zhang C, Kou R, Xie K, Song F (2019). Mitophagy protects against acetaminophen-induced acute liver injury in mice through inhibiting NLRP3 inflammasome activation. Biochem Pharm.

[26] Kim MJ, Kim WS, Kim DO, Byun JE, Huy H, Lee SY (2017). Macrophage migration inhibitory factor interacts with thioredoxin-interacting protein and induces NF-κB activity. Cell Signal.

[27] Rittirsch D, Huber-Lang MS, Flierl MA, Ward PA (2009). Immunodesign of experimental sepsis by cecal ligation and puncture. Nat Protoc.

[28] Al-Abed Y, Dabideen D, Aljabari B, Valster A, Messmer D, Ochani M (2005). ISO-1 binding to the tautomerase active site of MIF inhibits its pro-inflammatory activity and increases survival in severe sepsis. J Biol Chem.

[29] Peng X, Wang Y, Li H, Fan J, Shen J, Yu X (2019). ATG5-mediated autophagy suppresses NF-κB signaling to limit epithelial inflammatory response to kidney injury. Cell Death Dis.

[30] Shin HM, Kim MH, Kim BH, Jung SH, Kim YS, Park HJ (2004). Inhibitory action of novel aromatic diamine compound on lipopolysaccharide-induced nuclear translocation of NF-kappaB without affecting IkappaB degradation. FEBS Lett.

[31] Chen X, He WT, Hu L, Li J, Fang Y, Wang X (2016). Pyroptosis is driven by non-selective gasdermin-D pore and its morphology is different from MLKL channel-mediated necroptosis. Cell Res.

