# Supplementary material for: Downregulation of macrophage migration inhibitory factor attenuates NLRP3 inflammasome mediated pyroptosis in sepsis-induced AKI
Source: Cell Death Discov. 2022 Feb 14;8:61. doi: 10.1038/s41420-022-00859-z (PMC8844278; doi:10.1038/s41420-022-00859-z)
Supplement: Supplementary file 1 — Uncropped Western blots [file 41420_2022_859_MOESM1_ESM.pptx]

## Slide 1
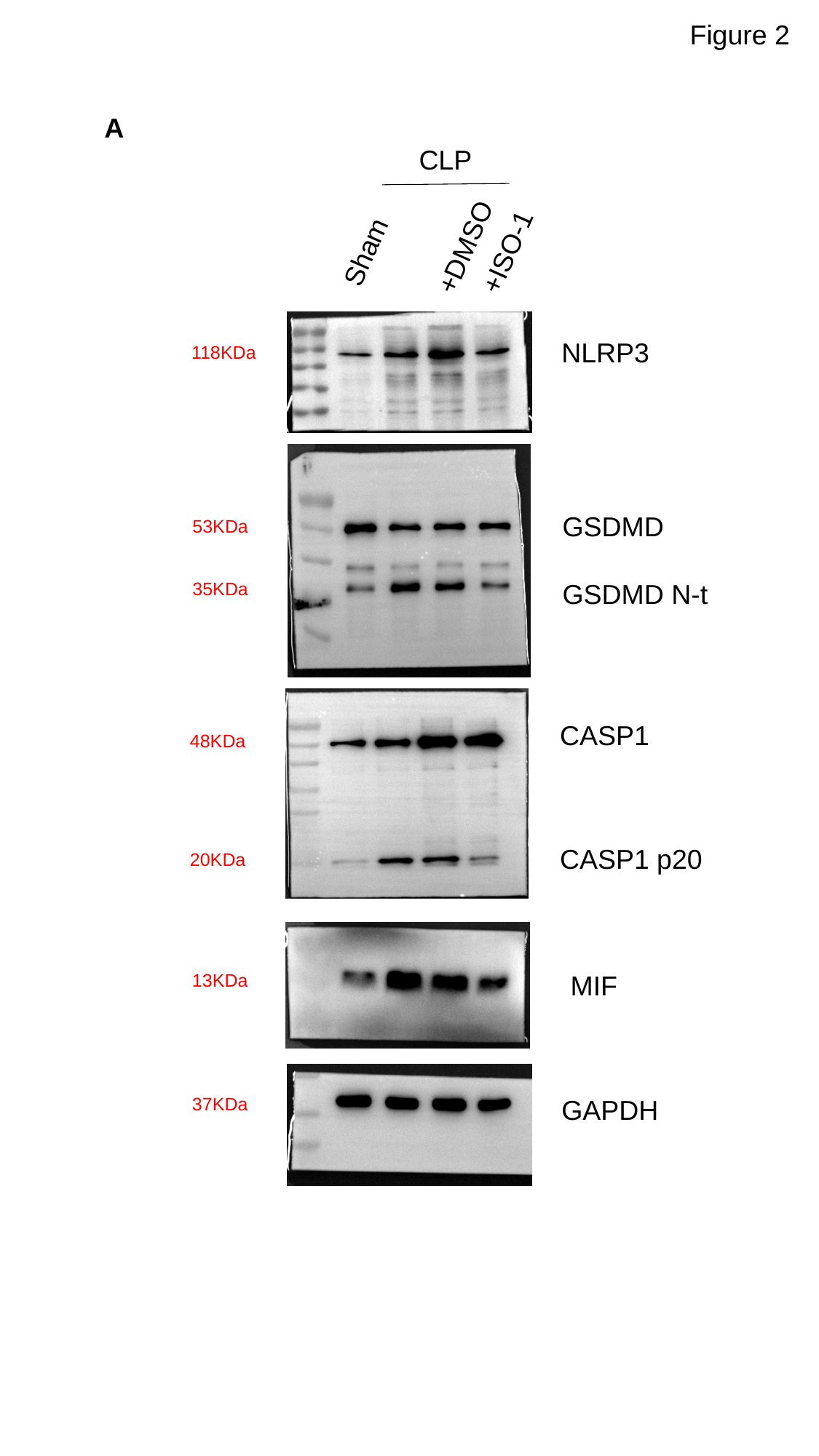

Figure 2
A
CLP
+DMSO
Sham
+ISO-1
NLRP3
118KDa
GSDMD
53KDa
35KDa
GSDMD N-t
CASP1
48KDa
CASP1 p20
20KDa
13KDa
MIF
37KDa
GAPDH

## Slide 2
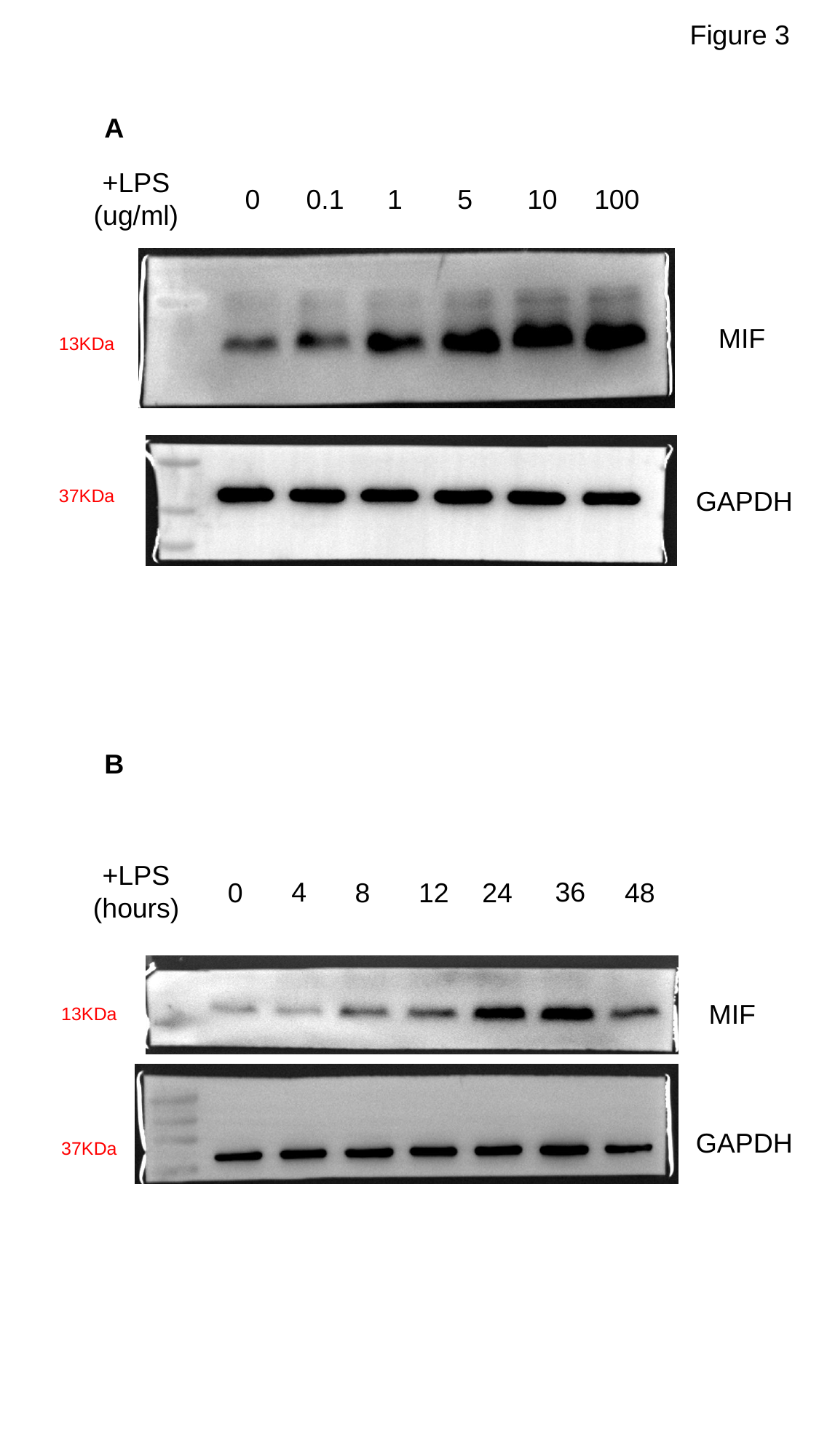

Figure 3
A
+LPS
(ug/ml)
0
0.1
1
5
10
100
MIF
13KDa
37KDa
GAPDH
B
+LPS
(hours)
4
36
0
8
12
24
48
MIF
13KDa
GAPDH
37KDa

## Slide 3
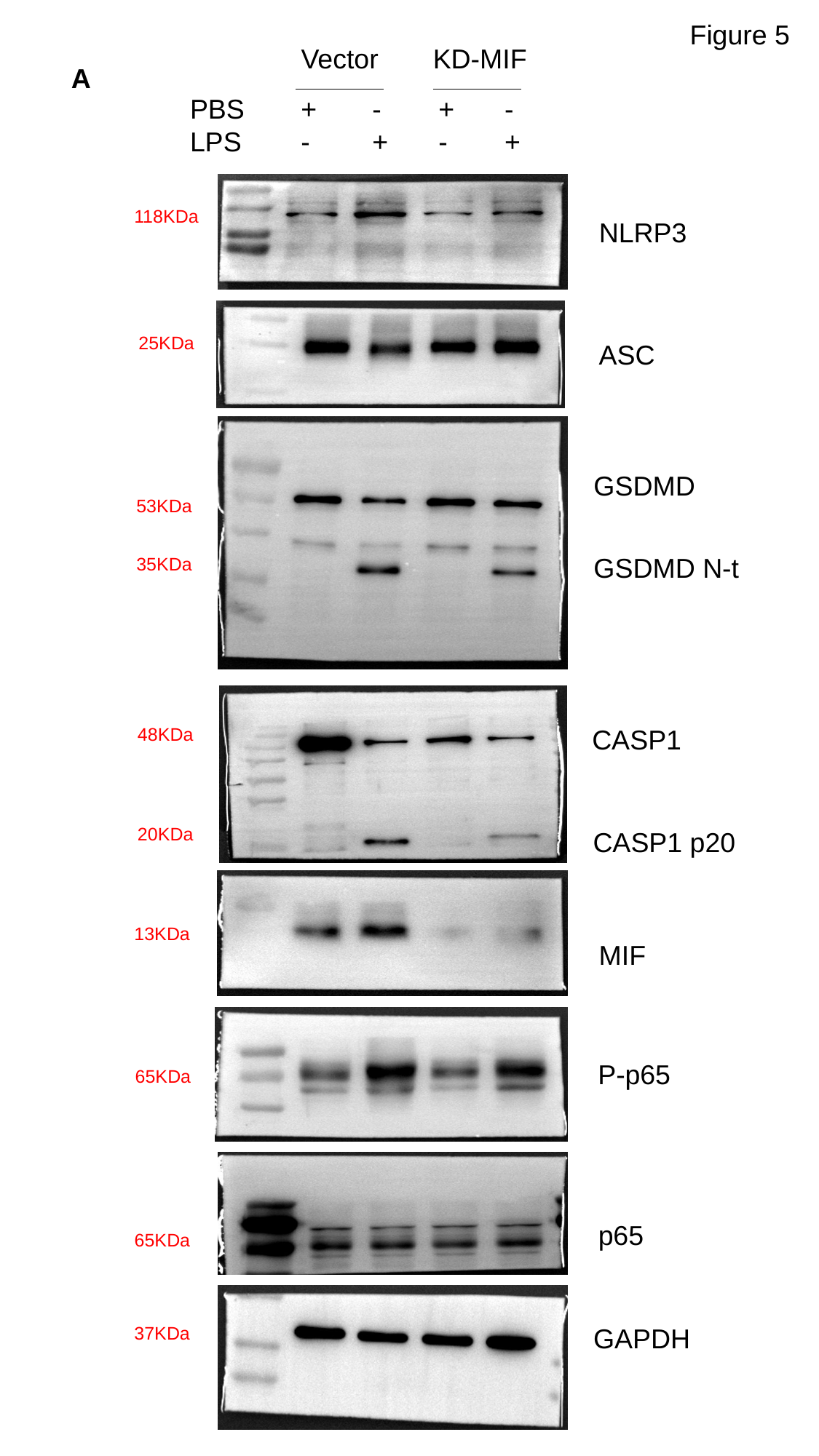

Figure 5
Vector
KD-MIF
A
PBS
LPS
+
-
-+
+
-
-+
118KDa
NLRP3
25KDa
ASC
GSDMD
53KDa
GSDMD N-t
35KDa
CASP1
48KDa
20KDa
CASP1 p20
13KDa
MIF
P-p65
65KDa
p65
65KDa
37KDa
GAPDH

## Slide 4
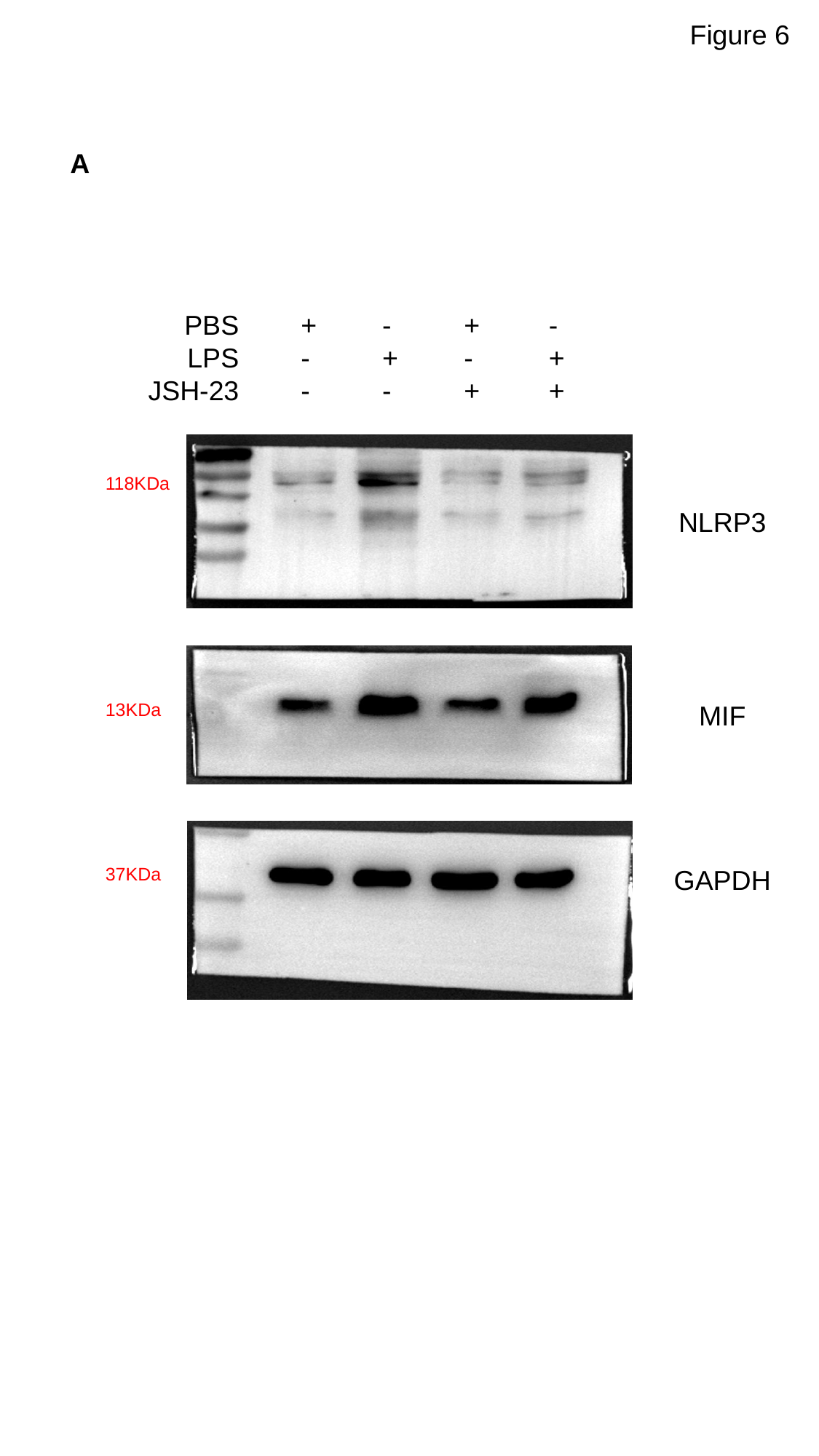

Figure 6
A
PBS
LPS
JSH-23
+
-
-
-
+
-
+
-
+
-
+
+
118KDa
NLRP3
13KDa
MIF
37KDa
GAPDH
